# Effect of Pixel Offset Adjustments for XY Plane Dimensional Compensation in Digital Light Processing 3D Printing on the Surface Trueness and Fit of Zirconia Crowns

**DOI:** 10.3390/jfb16030103

**Published:** 2025-03-14

**Authors:** KeunBaDa Son, Ji-Min Lee, Kyoung-Jun Jang, Sang-Kyu Lee, Jun Ho Hwang, Jong Hoon Lee, Hyun Deok Kim, So-Yeun Kim, Kyu-Bok Lee

**Affiliations:** 1 Advanced Dental Device Development Institute (A3DI), Kyungpook National University, Daegu 41940, Republic of Korea; oceanson@knu.ac.kr (K.S.); wlals9408@naver.com (J.-M.L.); 2 Department of Dental Science, Graduate School, Kyungpook National University, Daegu 41940, Republic of Korea; 3 3D Controls Co., Ltd., Gangseo-gu, Busan 46721, Republic of Korea; jangkj@3dcontrols.co.kr (K.-J.J.); leesk@3dcontrols.co.kr (S.-K.L.); 4 Institute of Advanced Convergence Technology, Kyungpook National University, Daegu 41061, Republic of Korea; hjh@iact.or.kr (J.H.H.); laser@knu.ac.kr (J.H.L.); 5 School of Electronics Engineering, Kyungpook National University, Daegu 41566, Republic of Korea; hdkim@knu.ac.kr; 6 Department of Prosthodontics, School of Dentistry, Kyungpook National University, Daegu 41940, Republic of Korea

**Keywords:** digital light processing 3D printing, zirconia crowns, pixel offset, marginal and internal fit, surface trueness

## Abstract

This study aimed to evaluate the effect of pixel offset adjustments in digital light processing (DLP) three-dimensional (3D) printing on the marginal and internal fit and surface trueness of zirconia crowns. Zirconia crowns were designed using dental computer-aided design software (Dentbird; Imagoworks) and fabricated with a vat photopolymerization DLP 3D printer (TD6+; 3D Controls) under three pixel offset conditions (−1, 0, and 1). Pixel offset refers to the controlled modification of the outermost pixels in the XY plane during printing to compensate for potential dimensional inaccuracies. The marginal and internal fit was assessed using a triple-scan protocol and quantified using root mean square (RMS) values. Surface trueness was evaluated by measuring RMS, positive and negative errors between the designed and fabricated crowns. Statistical analyses included one-way ANOVA and Pearson correlation analysis (α = 0.05). The Pixel offset had a significant effect on fit accuracy and surface trueness (*p* < 0.05). Higher pixel offsets increased marginal discrepancies (*p* = 0.004), with the marginal gap exceeding 120 µm at a pixel offset of 1 (114.5 ± 14.6 µm), while a pixel offset of −1 (85.5 ± 18.6 µm) remained within acceptable limits (*p* = 0.003). Surface trueness worsened with increasing pixel offset, showing greater positive errors (*p* < 0.001). Optimizing pixel offset in DLP 3D printing is crucial to ensuring clinically acceptable zirconia crowns. Improper settings may increase marginal discrepancies and surface errors, compromising restoration accuracy.

## 1. Introduction

The adoption of three-dimensional (3D) printing technology in digital dentistry has significantly enhanced the efficiency of fabrication workflows, reduced production time, and provided cost-effective solutions while maintaining high precision [1,2]. Consequently, 3D printing has become an integral part of dental applications, including the fabrication of prosthetic restorations, computer-guided surgical templates, and orthodontic appliances [3,4,5]. With continuous advancements in intraoral and extraoral scanning technologies, the utilization of 3D printing in dental computer-aided design and computer-aided manufacturing (CAD/CAM) systems continues to expand, enabling more accurate and customized treatment solutions [6].

Despite its widespread adoption, the primary applications of 3D printing in digital dentistry remain largely confined to polymer-based materials, such as those used for interim restorations, surgical guides, and orthodontic devices [7,8]. However, recent advancements in composite polymer-based 3D printing have demonstrated improved mechanical properties, broadening its clinical applicability [9,10]. Furthermore, the development of ceramic-based 3D printing technologies has attracted considerable interest due to its potential advantages over conventional subtractive manufacturing, such as minimizing material waste and reducing wear on milling burs [10,11]. Nevertheless, ceramic 3D printing presents unique challenges, including the high viscosity of ceramic slurries, the heterogeneity of composite materials, and the necessity for extensive post-processing steps such as debinding and sintering [12,13,14]. These factors directly impact the dimensional accuracy of dental restorations, emphasizing the need for systematic investigations to optimize fabrication parameters [15]. Compared to polymer-based 3D printing, ceramic 3D printing involves more complex and less controllable variables, requiring further research to ensure precision in dental prosthesis fabrication. In particular, volumetric changes occurring during the debinding and sintering stages can induce shrinkage or expansion [16,17], necessitating strategies to compensate for these dimensional alterations.

Among various 3D printing techniques utilized in digital dentistry, vat photopolymerization is one of the most extensively applied methods, wherein light-sensitive resins are selectively cured layer by layer [18,19]. Stereolithography (SLA) technology employs a laser to achieve high-resolution fabrication but requires extended production time [20]. In contrast, digital light processing (DLP) utilizes a digital micromirror device to project entire layers simultaneously, offering enhanced efficiency and cost-effectiveness compared to SLA [21,22]. In DLP printing, the pixel size of the projected image determines the resolution of the XY plane, and modifying the pixel offset can enhance dimensional accuracy [23,24,25]. Additionally, adjusting the pixel offset can compensate for the material shrinkage or expansion that occurs during fabrication, which varies depending on the printing material, such as polymers, ceramics, and metals [26].

Zirconia-based 3D printing has gained particular interest in digital dentistry due to zirconia’s excellent biocompatibility, high mechanical strength, and favorable esthetic properties [27,28]. However, the clinical feasibility of zirconia 3D printing is still under investigation, particularly concerning fabrication accuracy, marginal and internal fit, surface trueness, and long-term clinical performance [29,30]. Given that the volumetric changes induced by debinding and sintering can significantly affect the final prosthetic dimensions [31,32], optimizing fabrication parameters to compensate for these changes remains a crucial research objective. In DLP 3D printing, pixel offset serves as a key parameter for compensating dimensional inaccuracies by modifying the external and internal boundaries of a printed object in the XY plane [23,24,25]. Given that ceramic 3D printing is particularly susceptible to volumetric shrinkage during the debinding and sintering processes, precise pixel offset adjustments are essential to maintaining fabrication accuracy [26,29]. However, limited studies have systematically investigated the quantitative effects of different pixel offset values on zirconia crown adaptation. In this study, three pixel offset conditions were evaluated (−1, 0, and 1) to assess their impact on marginal and internal fit, as well as surface trueness. The pixel offset of 0 was chosen as the control, representing a default setting without dimensional modifications. The pixel offset of −1 was selected to evaluate the effect of contracting the printed structure, potentially improving internal adaptation, whereas the pixel offset of 1 was included to assess the influence of expansion on marginal and internal discrepancies. These values were determined based on the resolution constraints of the DLP system, which limits offset adjustments to whole pixel increments of 72 µm. By investigating these conditions, this study aims to establish evidence-based guidelines for optimizing pixel offset parameters in 3D-printed zirconia restorations.

Therefore, this study aimed to evaluate the effect of pixel offset adjustments in DLP 3D printing on the marginal and internal fit and surface trueness of zirconia crowns. Specifically, the study investigates whether modifying the pixel offset in the XY plane can mitigate dimensional inaccuracies introduced during the printing and post-processing stages. The null hypothesis of this study was that there are no significant differences in the marginal and internal fit and surface trueness among zirconia crowns fabricated with different pixel offset settings: 0 (no adjustment), +1 (expanding the external and internal contours by one pixel), and −1 (contracting the external and internal contours by one pixel).

## 2. Materials and Methods

### 2.1. Sample Preparation

**Figure 1:**
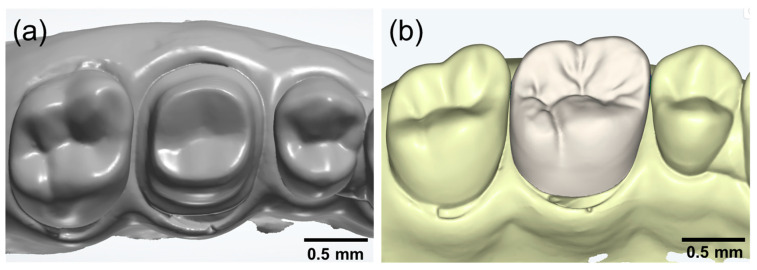
Dental typodont model used in this study. (**a**) Digitized working cast obtained through intraoral scanning. (**b**) Designed crown.

Tooth preparation for a zirconia crown was performed on a maxillary right first molar typodont model (D85DP-500B.1; Nissin Dental, Tokyo, Japan) (Figure 1). The preparation included a 1.5 mm axial and occlusal reduction, a chamfer finish line with a margin width of 1.0 mm, and a supragingival placement of the finish line (Figure 1).

Following preparation, the maxillary right first molar and adjacent teeth were digitized using an intraoral scanner (i700; MEDIT, Seoul, Republic of Korea) to generate a virtual cast (Figure 1). Additional scans of the opposing dentition and occlusal records were obtained to ensure proper occlusal alignment.

The zirconia crown was digitally designed using dental CAD software (Dentbird; Imagoworks, Seoul, Republic of Korea), specifically configured for zirconia crown fabrication (Figure 1). The cement space was set at 50 µm, and the crown morphology was generated using an AI-based design feature. The final CAD model was reviewed and verified by an experienced professional. The design parameters were as follows: cement space—50 µm; adaptive extra gap—0 µm; height for minimal gap—50 µm; and margin width—100 µm.

**Figure 2:**
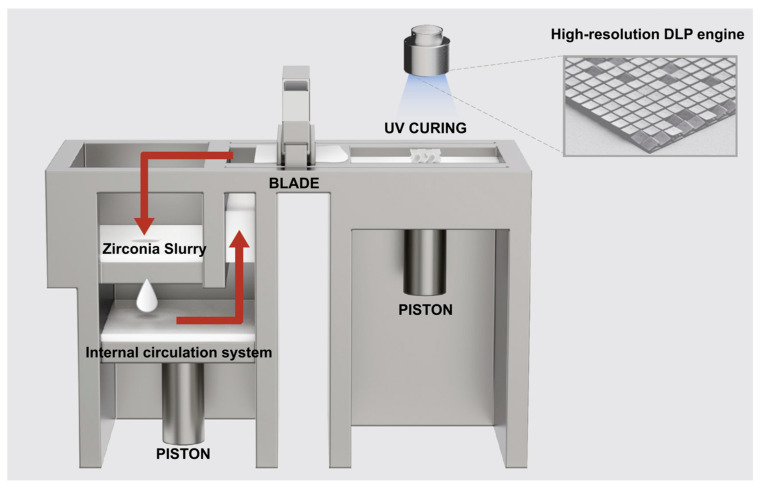
Schematic representation of the DLP 3D printing system used in this study. The arrows indicate the flow of the zirconia slurry within the internal circulation system, which ensures a consistent material supply for the printing process. (The arrows in the figure indicate the flow of the zirconia slurry within the internal circulation system).

Crown fabrication was performed using a DLP 3D printer (TD6+; 3D Controls, Busan, Republic of Korea) and a zirconia material (3DCERA; 3D Controls, Busan, Republic of Korea) (Table 1) (Figure 2). The DLP 3D printer utilizes vat photopolymerization technology with a 1920 × 1080 resolution projection system and a pixel pitch of 72 µm in the XY plane. The build size is 120 × 68 × 45 mm (green body) and the layer thickness was set to 25 µm. A dual-blade system was used for uniform layer flattening, optimizing printing accuracy. The printing data were processed using a slicer software program (3D Controls Slicer; 3D Controls, Busan, Republic of Korea), which generated cross-sectional layers in the XY plane before fabrication.

To evaluate the effect of pixel offset adjustments, three experimental groups were established: Offset −1, Offset 0, and Offset 1. In DLP 3D printing, each XY cross-section of the crown consists of two boundary lines: an external surface contour line and an internal surface contour line that define the outer and inner geometry of the structure. Pixel offset refers to the intentional modification of these boundary lines during slicing to compensate for potential printing inaccuracies. In the Offset −1 group, both the external and internal boundary lines were shifted inward by one pixel width (72 µm) relative to the original design. This resulted in an overall reduction in the printed crown dimensions. In the Offset 1 group, both boundary lines were shifted outward by one pixel width (72 µm), effectively increasing the final printed dimensions. In the Offset 0 group, no modifications were applied, maintaining the boundary lines as per the original CAD model. Intermediate values (e.g., −0.5, 0.5) could not be applied as offset parameters due to the inherent characteristics of DLP technology. In this system, photopolymerization is induced by selectively projecting light in discrete pixel units, allowing adjustments only in whole-pixel increments. Consequently, fractional offset values cannot be implemented within the slicing and printing process.

**Figure 3:**
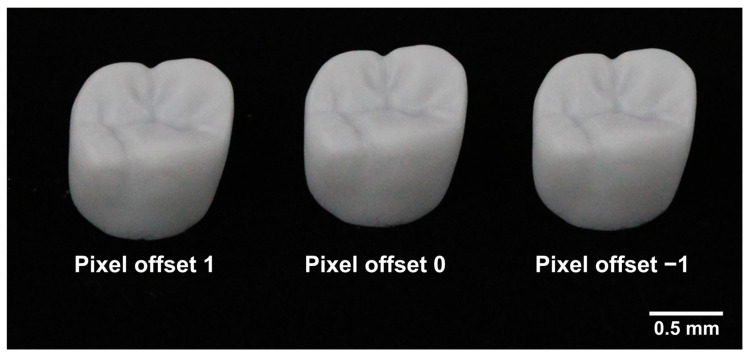
Zirconia crowns fabricated using DLP 3D printing with different pixel offset settings.

The 3D printer used in this study had a pixel size of 72 µm in the XY plane, and the layer thickness in the Z-direction was set to 25 µm. The build orientation was set to 0°, with the occlusal surface positioned parallel to the printing platform. A total of 30 crowns were fabricated (N = 10 per group) (Figure 3). Following printing, the crowns underwent cleaning, debinding, and sintering for post-processing. The cleaning process was performed using a cleaner (C1; 3D Controls, Busan, Republic of Korea), which operates with a direct injection nozzle system to minimize damage to the green body. For the sintering process, a furnace (R0; 3D Controls, Busan, Republic of Korea) was used, capable of reaching a maximum temperature of 1600 °C, ensuring proper densification of the zirconia crowns.

The required sample size was determined through a priori power analysis using one-way ANOVA with three independent groups (*k* = 3). A pilot experiment conducted under the same methodology as this study yielded an estimated effect size (f = 0.8775). The power analysis was performed using a power software program (G*Power version 3.1.9.7; Heinrich-Heine-Universität Düsseldorf, Germany) with a significance level (α) of 0.05 and a statistical power of 0.98 (actual power = 0.9874). The results indicated that a minimum of 10 samples per group was required to achieve adequate statistical power. Consequently, 10 crowns per group were fabricated, resulting in a total sample size of 30 (N = 30 in total).

### 2.2. Evaluation of Marginal and Internal Fit

**Figure 4:**
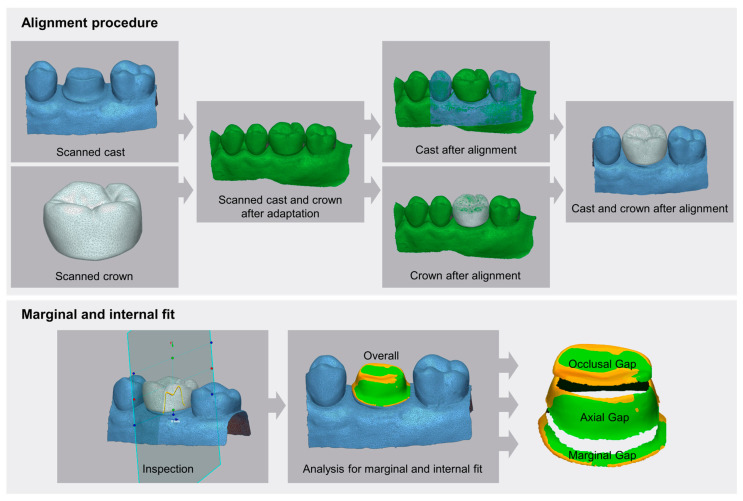
Evaluation method for marginal and internal fit analysis. The alignment procedure was performed using the scanned cast and crown in their adapted state. The arrows indicate the sequential steps in the alignment process, where the scanned cast and crown were used as reference data for alignment. The gap between the intaglio surface of the aligned crown and the cast was analyzed across all corresponding point cloud data. (The arrows in the figure illustrate the step-by-step procedure of the alignment process and marginal/internal fit analysis).

The marginal and internal fit of the zirconia crowns was evaluated using a triple-scan protocol (Figure 4). First, the intaglio and occlusal surfaces of the fabricated crowns were scanned using a laboratory scanner (E1; 3Shape, Copenhagen, Denmark) (Figure 4). Second, the typodont model (D85DP-500B.1; Nissin Dental, Tokyo, Japan) was scanned using the same laboratory scanner (Figure 4). Third, the crown was seated on the typodont model, and the assembled structure was scanned to obtain the final dataset (Figure 4). The crown adaptation process was performed by an experienced operator to ensure accurate seating before scanning.

The acquired scan data were processed and aligned using 3D inspection software (Geomagic Control X; Version 2017.0.1; 3D Systems, Rock Hill, SC, USA) (Figure 4). The alignment procedure consisted of an initial registration step, followed by a best-fit alignment, with the final assembled scan serving as the reference for aligning the individual scans of the crown and typodont model (Figure 4). The alignment error for the best-fit alignment process was analyzed to be below 3.1 µm, ensuring precise superimposition of the scanned datasets. Finally, sectional views were inspected to verify whether appropriate marginal and internal gaps were maintained after the alignment process (Figure 4).

The marginal and internal fit were quantitatively assessed using the root mean square (RMS) of the mean distances between the intaglio surface of the crown and the prepared tooth surface of the typodont model. The RMS value was calculated using the following Equation (1):

(1) $$RMS=\frac{1}{\sqrt{n}}\cdot \sqrt{\sum_{i=1}^{n}{\left(X_{1,i}-X_{2,i}\right)}^{2}}$$

where $X_{1,i}$ represents the *i*-th measurement point on the intaglio surface of the crown, and $X_{2,i}$ represents the corresponding measurement point on the prepared tooth surface. The RMS value quantifies the mean absolute deviation between these two surfaces, providing an objective measure of the crown’s adaptation.

To facilitate visual interpretation, a color map analysis was performed (Figure 4). The color scale was set to represent the gap distribution, with green areas indicating gaps within 100 µm, which are considered clinically acceptable. Warmer colors (yellow to red) indicate increasing positive deviations, reaching up to 400 µm, signifying excessive gaps, while cooler colors (blue to dark blue) represent negative deviations, corresponding to areas where the crown exhibited excessive internal adaptation.

For a more detailed assessment, the marginal and internal fit evaluation was categorized into four distinct regions: overall fit, occlusal gap, axial gap, and marginal gap. The marginal gap was defined as the area extending 1.0 mm above the finish line, representing the adaptation along the crown margin (Figure 4). The axial gap referred to the vertical walls of the prepared tooth, evaluating the crown’s adaptation along the axial surfaces (Figure 4). The occlusal gap measured the discrepancies at the occlusal contacts between the crown and the prepared tooth (Figure 4). The overall fit encompassed all these regions to provide a comprehensive evaluation of the adaptation accuracy (Figure 4).

### 2.3. Evaluation of Surface Trueness

**Figure 5:**
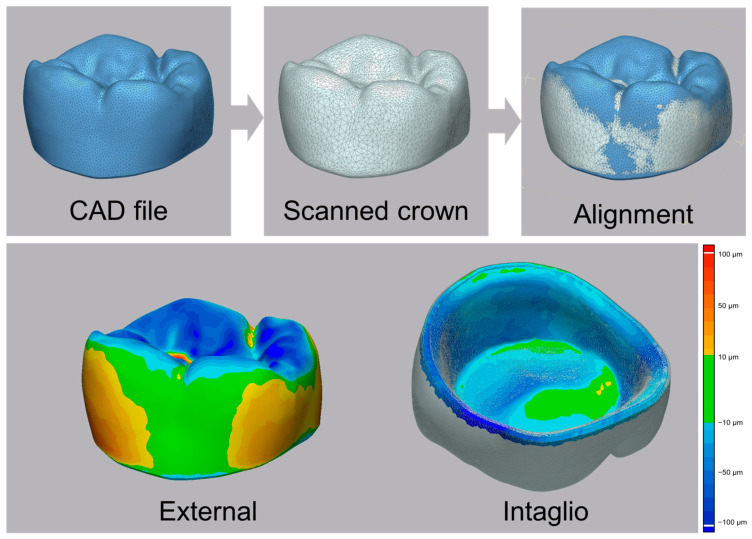
Evaluation method for surface trueness analysis. The designed crown model and the scanned crown were aligned, and deviations between corresponding point cloud data were quantified to assess surface trueness.

The surface trueness of the fabricated zirconia crowns was evaluated by comparing the designed CAD model with the scanned crowns obtained using a laboratory scanner (E1; 3Shape, Copenhagen, Denmark) (Figure 5). The evaluation process consisted of three steps: (1) scanning the CAD-designed crown to establish a reference model, (2) scanning the fabricated crowns after post-processing, and (3) aligning the scanned data with the CAD reference model for deviation analysis (Figure 5).

The alignment of the scanned crowns with the CAD reference model was conducted using a best-fit registration method in 3D inspection software (Geomagic Control X; Version 2017.0.1; 3D Systems, Rock Hill, SC, USA) (Figure 5). The analysis was performed separately for the external and intaglio surfaces to evaluate fabrication accuracy (Figure 5).

To quantify surface deviations, three key parameters were calculated: RMS deviation, mean positive error, and mean negative error. The RMS value represents the overall deviation magnitude, irrespective of direction, and was calculated using the following Equation (2):

(2) $$RMS=\frac{1}{\sqrt{n}}\cdot \sqrt{\sum_{i=1}^{n}{D_{i}}^{2}}$$

where $D_{i}$ represents the deviation at each measurement point, and *n* is the total number of evaluated points. Therefore, the RMS value serves as a quantitative measure of the fabrication accuracy of the crown, with lower values indicating superior surface trueness and higher dimensional precision.

Additionally, mean positive and negative errors were separately analyzed to distinguish between overbuilt and underbuilt regions. The mean positive error indicates that the fabricated crown surface was positioned outward relative to the CAD design, meaning the actual crown was larger than intended. In contrast, the mean negative error signifies inward displacement, indicating that the fabricated surface was undersized compared to the design.

A color-coded deviation map was applied to visualize the deviations (Figure 5). The color scale was set with green representing deviations within ±10 µm, yellow to red indicating positive deviations up to 100 µm, and blue to dark blue denoting negative deviations up to −100 µm. This classification allowed for precise evaluation of the manufacturing accuracy of the zirconia crowns fabricated using DLP 3D printing.

### 2.4. Statistical Analysis

All statistical analyses were conducted using statistical software (SPSS v29.0; IBM Corp., USA), with the significance level set at α = 0.05. The Shapiro–Wilk test was employed to assess the normality of data distribution. Comparisons of marginal and internal fit and surface trueness among the three pixel offset groups (−1, 0, and 1) were performed using one-way analysis of variance (ANOVA), followed by Tukey’s honest significant difference (HSD) test for post hoc pairwise comparisons.

To evaluate the relationship between intaglio surface trueness and marginal and internal fit, Pearson correlation coefficient (PCC) analysis was conducted. The correlation strength was classified as strong (correlation coefficient = 0.7–0.9), moderate (correlation coefficient = 0.4–0.6), or weak (correlation coefficient = 0.1–0.3). A *p*-value of less than 0.05 was considered statistically significant for all analyses.

## 3. Results

### 3.1. Result of Marginal and Internal Fit

The effect of pixel offset adjustment on the marginal and internal fit of zirconia crowns fabricated using DLP 3D printing was evaluated, demonstrating statistically significant differences based on the pixel offset values (Table 2; *p* < 0.05). The mean overall fit values were 86.5 ± 17.4 µm at a pixel offset of −1, 96.0 ± 15.1 µm at 0, and 109.9 ± 9.2 µm at 1. As the pixel offset increased, the overall fit decreased, with a statistically significant difference observed between pixel offsets of −1 and 1 (Table 2; *p* = 0.004). Significant differences were also observed in the marginal and occlusal gaps (Table 2; *p* = 0.003 and *p* < 0.001, respectively). The marginal gap was smallest at a pixel offset of −1 (85.5 ± 18.6 µm) and largest at 1 (114.5 ± 14.6 µm), with a statistically significant difference between these two groups. Similarly, the occlusal gap was lowest at a pixel offset of −1 (80.3 ± 28.4 µm) and highest at 1 (141.9 ± 20.2 µm), with statistically significant differences among all groups (Table 2). However, no statistically significant differences were observed in the axial gap among the different pixel offset values (Table 2; *p* = 0.258).

**Figure 6:**
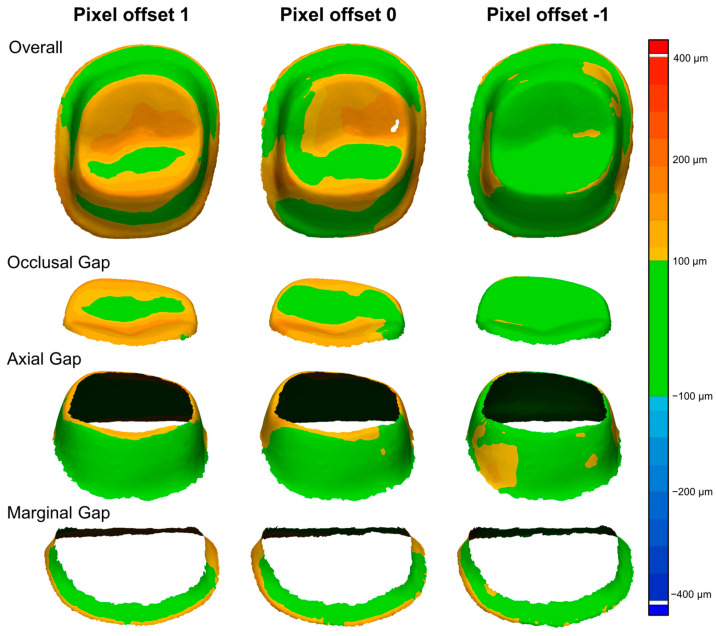
Comparison of color maps for marginal and internal fit. The color scale on the right represents the gap distribution, with green areas indicating gaps within 100 µm, while warmer colors (yellow to red) represent increasing deviations, reaching up to 400 µm.

Figure 6 shows the color maps of marginal and internal fit for zirconia crowns fabricated using different pixel offset conditions (−1, 0, and 1). The pixel offset of −1 exhibited the largest green areas, indicating gaps within 100 µm, while the pixel offset of 1 showed more yellow and orange regions, representing increased discrepancies. The marginal and occlusal gaps were visibly larger at a pixel offset of 1, whereas the axial gap showed minimal variation across conditions.

### 3.2. Result of Surface Trueness

The effect of pixel offset adjustment on the RMS value of surface trueness in zirconia crowns fabricated using DLP 3D printing was evaluated, revealing statistically significant differences among pixel offset values (Table 3; *p* < 0.001). The mean RMS surface trueness value for the overall region was highest at a pixel offset of 1 (57.8 ± 5.2 µm), which was significantly greater than those observed at pixel offsets of 0 (32.8 ± 2.9 µm) and −1 (36.3 ± 4.3 µm) (Table 3; *p* < 0.001). For the external surface, the highest RMS surface trueness value was recorded at a pixel offset of 1 (61.8 ± 4.7 µm), with statistically significant differences compared to pixel offsets of 0 (35.9 ± 3.7 µm) and −1 (34.3 ± 4.6 µm) (Table 3; *p* < 0.001). In the intaglio surface region, the RMS surface trueness value was highest at a pixel offset of 1 (49.5 ± 6.6 µm), with statistically significant differences compared to pixel offsets of 0 (26.0 ± 3.1 µm) and −1 (39.6 ± 4.5 µm) (Table 3; *p* < 0.001).

**Figure 7:**
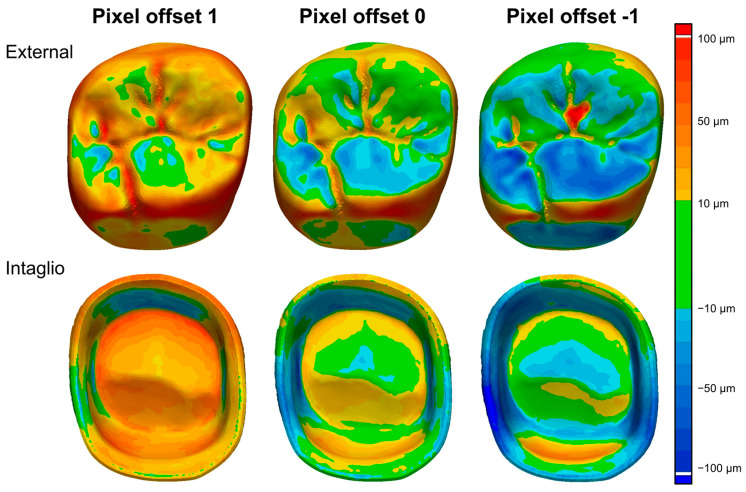
Comparison of color maps for surface trueness. The color scale on the right represents surface deviations, with green areas indicating deviations within ±10 µm, while warmer colors (yellow to red) represent increasing positive deviations, reaching up to 100 µm. Conversely, cooler colors (blue to dark blue) indicate negative deviations, with a maximum of −100 µm.

Figure 7 presents the color maps of surface trueness for zirconia crowns fabricated using different pixel offset conditions (−1, 0, and 1). The overall surface trueness deviations were highest at a pixel offset of 1, with extensive yellow and red regions indicating greater positive deviations, while the pixel offset of −1 showed increased blue areas, representing larger negative deviations. These trends align with the RMS surface trueness values, where the pixel offset of 1 exhibited the highest deviations (57.8 ± 5.2 µm overall, 61.8 ± 4.7 µm external, and 49.5 ± 6.6 µm intaglio), while the pixel offset of −1 displayed lower values with a greater proportion of negative errors.

The effect of pixel offset adjustment on the positive error value of surface trueness in zirconia crowns fabricated using DLP 3D printing was evaluated, revealing statistically significant differences among pixel offset values (Table 4; *p* < 0.001). The mean positive error value for the overall region was highest at a pixel offset of 1 (53.5 ± 5.1 µm), with statistically significant differences compared to pixel offsets of 0 (29.5 ± 2.7 µm) and −1 (23.6 ± 3.0 µm) (Table 4; *p* < 0.001). For the external surface, the highest positive error value was observed at a pixel offset of 1 (56.9 ± 4.6 µm), with statistically significant differences compared to pixel offsets of 0 (32.5 ± 3.1 µm) and −1 (24.9 ± 3.4 µm) (Table 4; *p* < 0.001). In the intaglio surface region, the highest positive error value was recorded at a pixel offset of 1 (46.6 ± 6.4 µm), which was significantly greater than those at pixel offsets of 0 (22.4 ± 3.3 µm) and −1 (19.1 ± 1.9 µm) (Table 4; *p* < 0.001).

The positive error distribution varied across pixel offset conditions, with the pixel offset of 1 exhibiting the most extensive yellow and red areas on both the external and intaglio surfaces (Figure 7). The highest positive error values were observed at a pixel offset of 1 (53.5 ± 5.1 µm overall, 56.9 ± 4.6 µm external, and 46.6 ± 6.4 µm intaglio), whereas the pixel offset of −1 showed significantly lower positive deviations (23.6 ± 3.0 µm overall, 24.9 ± 3.4 µm external, and 19.1 ± 1.9 µm intaglio) (Figure 7). This corresponds with the color map, where the pixel offset of −1 exhibited fewer high-deviation regions compared to 1 (Figure 7).

The effect of pixel offset adjustment on the negative error value of surface trueness in zirconia crowns fabricated using DLP 3D printing was evaluated, revealing statistically significant differences among pixel offset values (Table 5; *p* < 0.001). The mean negative error value for the overall region was highest at a pixel offset of −1 (32.1 ± 3.9 µm), with statistically significant differences compared to pixel offsets of 0 (18.2 ± 2.6 µm) and 1 (15.6 ± 3.4 µm) (Table 5; *p* < 0.001). For the external surface, the highest negative error value was observed at a pixel offset of −1 (28.7 ± 4.3 µm), with statistically significant differences compared to pixel offsets of 0 (17.2 ± 2.7 µm) and 1 (12.9 ± 4.0 µm) (Table 5; *p* < 0.001). In the intaglio surface region, the highest negative error value was recorded at a pixel offset of −1 (36.6 ± 4.0 µm), which was significantly greater than those at pixel offsets of 0 (19.2 ± 3.4 µm) and 1 (17.1 ± 4.8 µm) (Table 5; *p* < 0.001).

The negative error distribution was most pronounced at a pixel offset of −1, as indicated by the increased presence of blue-shaded regions, particularly on the intaglio surface (Figure 7). The largest negative errors were observed at a pixel offset of −1 (32.1 ± 3.9 µm overall, 28.7 ± 4.3 µm external, and 36.6 ± 4.0 µm intaglio), whereas the pixel offset of 1 had the smallest negative errors (Figure 7). The pixel offset of 0 displayed an intermediate distribution, with both positive and negative deviations present in the color maps (Figure 7).

### 3.3. Result of Correlation Between Intaglio Surface Trueness and Marginal and Internal Fit

**Figure 8:**
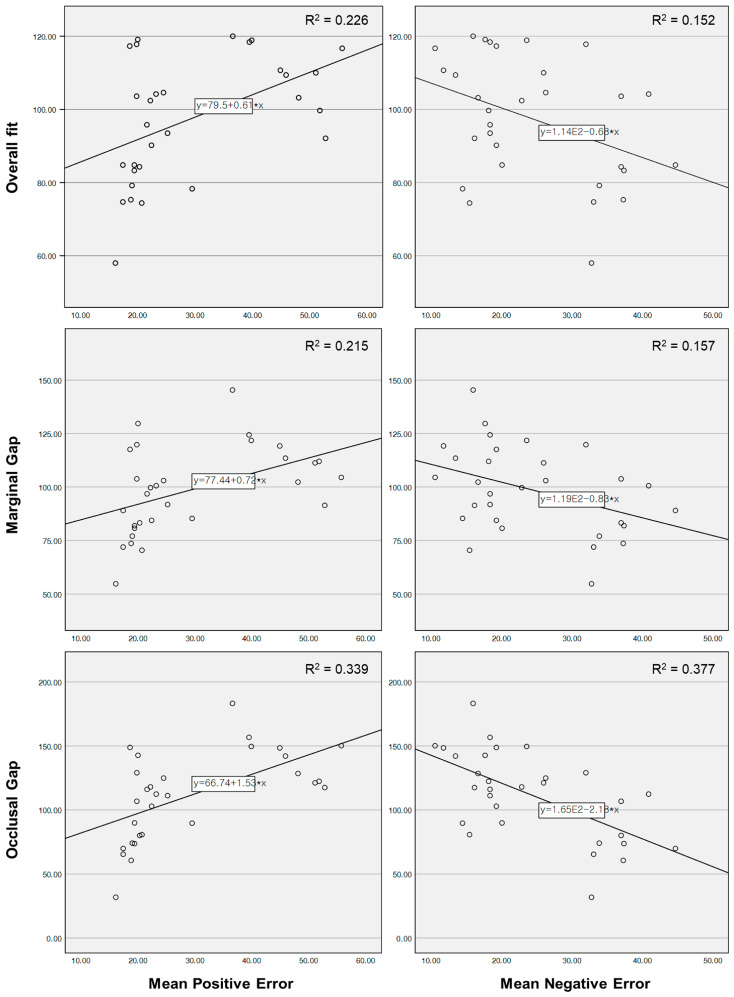
Correlation between intaglio surface trueness and marginal and internal fit. Positive intaglio errors exhibit a direct correlation with increased marginal and internal discrepancies, whereas negative intaglio errors demonstrate an inverse correlation, indicating improved fit accuracy.

The correlation between intaglio surface trueness parameters and marginal and internal fit parameters was analyzed, revealing statistically significant relationships for specific variables (Table 6; *p* < 0.05). A moderate positive correlation was observed between overall fit and mean positive error (Table 6; Figure 8; PCC = 0.475, *p* = 0.008), whereas overall fit exhibited a weak negative correlation with mean negative error (Table 6; Figure 8; PCC = −0.390, *p* = 0.033). Similarly, the marginal gap showed a moderate positive correlation with mean positive error (Table 6; Figure 8; PCC = 0.464, *p* = 0.01) and a weak negative correlation with mean negative error (Table 6; Figure 8; PCC = −0.396, *p* = 0.03). No statistically significant correlations were identified between axial gap and any of the surface trueness parameters (Table 6; *p* > 0.05). In contrast, the occlusal gap demonstrated a moderate positive correlation with mean positive error (Table 6; Figure 8; PCC = 0.582, *p* < 0.001) and a strong negative correlation with mean negative error (Table 6; Figure 8; PCC = −0.614, *p* < 0.001).

## 4. Discussion

This study evaluated the effect of pixel offset adjustments on DLP 3D printing on the marginal and internal fit as well as the surface trueness of zirconia crowns. The null hypothesis, which posited no significant differences among the pixel offset conditions (−1, 0, and 1), was rejected, as the results demonstrated that pixel offset significantly influenced both fit accuracy and surface deviations (*p* < 0.05). Increasing the pixel offset led to greater marginal discrepancies, with a pixel offset of 1 yielding a marginal gap of 114.5 ± 14.6 µm, approaching the clinically unacceptable threshold of 120 µm, whereas a pixel offset of −1 maintained the marginal gap (85.5 ± 18.6 µm) within clinically acceptable limits. Furthermore, surface trueness deteriorated with increasing pixel offset, as indicated by greater positive deviations, while lower pixel offsets resulted in increased negative deviations. Correlation analysis revealed that higher positive internal deviations were associated with poorer fit accuracy, whereas greater negative deviations contributed to improved adaptation. These findings underscore the critical importance of optimizing pixel offset in DLP 3D printing to achieve clinically acceptable zirconia restorations. Failure to appropriately calibrate the pixel offset may result in excessive marginal discrepancies and surface inaccuracies, potentially compromising the clinical longevity and functional reliability of digitally fabricated prostheses. These results can be attributed to the inherent characteristics of ceramic materials in 3D printing, particularly the debinding and sintering processes. Following 3D printing, ceramic materials must undergo debinding and sintering to achieve their final properties [16,17]. The debinding process involves the removal of organic binders that hold the ceramic particles together, while the sintering process facilitates densification by fusing the ceramic particles at high temperatures [18]. These processes inevitably lead to volumetric shrinkage, which can impact the dimensional accuracy of the final restoration. Since dental crowns are designed to fit over prepared teeth, any volumetric shrinkage can naturally result in discrepancies between the crown and the underlying tooth structure, thereby affecting marginal and internal adaptation [31,32].

To compensate for this shrinkage, this study explored the application of pixel offset adjustments as a means of mitigating dimensional inaccuracies induced during the fabrication and post-processing stages. Previous research has highlighted the impact of volumetric changes in zirconia restorations, emphasizing the need for compensatory strategies during 3D printing [29,30]. By strategically modifying the pixel offset values, it is possible to counteract the effects of material shrinkage, thereby improving the overall fit accuracy and clinical feasibility of 3D-printed zirconia crowns. In particular, controlling pixel offset in the XY plane has been proposed as a viable method for enhancing dimensional stability in additively manufactured dental ceramics [26]. These findings align with prior studies suggesting that adjusting pixel resolution and calibration settings can influence the accuracy of 3D-printed structures [23,24,25]. Overall, this study contributes to the growing body of research on optimizing fabrication parameters for digital prosthodontics, reinforcing the importance of pixel offset adjustments in improving the fit and trueness of 3D-printed zirconia crowns.

### 4.1. Marginal and Internal Fit

This study evaluated the marginal and internal fit of zirconia crowns fabricated using DLP 3D printing with different pixel offset adjustments (−1, 0, and 1). The results demonstrated a significant increase in the marginal gap with increasing pixel offset values (*p* = 0.003). The smallest marginal gap was observed at a pixel offset of −1 (85.5 ± 18.6 µm), whereas the largest gap was recorded at a pixel offset of 1 (114.5 ± 14.6 µm). Similarly, overall internal fit showed an increasing trend as pixel offset values increased (*p* = 0.004). The most notable variation was observed in the occlusal gap, which measured 80.3 ± 28.4 µm at a pixel offset of −1 and increased to 141.9 ± 20.2 µm at a pixel offset of 1. However, no statistically significant differences were found in the axial gap among the different pixel offset groups (*p* = 0.258). These findings align with previous studies reporting that the fit of 3D-printed zirconia crowns is influenced by printing techniques and post-processing procedures [33]. Prior studies have shown that SLA-manufactured zirconia crowns exhibit marginal gaps in the range of 90 to 110 µm [34,35], comparable to the marginal fit observed in the present study for the pixel offset of −1. Additionally, studies comparing conventionally milled and 3D-printed crowns have suggested that 3D-printed crowns generally demonstrate superior internal adaptation. However, limited research has systematically examined the impact of pixel offset adjustments on the optimization of marginal and internal fit.

The axial gap plays a crucial role in the retention and durability of fixed dental prostheses, as it directly affects crown stability. The absence of a significant difference in axial gap among different pixel offset groups in this study may be attributed to the inherent seating behavior of fixed prostheses on prepared tooth structures. During crown placement, the axial walls make initial contact with the prepared tooth, and despite variations in pixel offset, the crown eventually settles into a position where the axial walls achieve close adaptation [36]. This mechanism likely explains why pixel offset adjustments significantly affected the marginal and occlusal gaps but not the axial gap in the present study.

Previous research has indicated that a marginal gap of up to 120 µm is clinically acceptable [37,38,39]. Numerous studies have proposed this threshold as a guideline for clinical feasibility, and it remains widely adopted [37,38,39]. Internal fit has been reported to be clinically acceptable within a range of 50 to 200 µm [40,41], with ideal occlusal gaps maintained below 70 µm [42]. In the present study, a pixel offset of −1 resulted in a marginal gap (85.5 ± 18.6 µm) and an occlusal gap (80.3 ± 28.4 µm) that remained within clinically acceptable limits. In contrast, a pixel offset of 1 yielded a marginal gap (114.5 ± 14.6 µm) approaching the upper limit of clinical acceptability, and an occlusal gap (141.9 ± 20.2 µm) exceeding the ideal range suggested by some studies [37,38,39,40,41,42]. These findings emphasize the importance of pixel offset adjustments in optimizing the fit of 3D-printed zirconia crowns, with a pixel offset of −1 appearing to provide the most favorable clinical outcomes.

Pixel offset is a key parameter in DLP 3D printing that affects printing resolution by modulating dimensional accuracy in the XY plane. Increasing the pixel offset in a positive direction (1) expands both the external and internal contour lines of the crown, resulting in overall enlargement of crown dimensions and a reduction in intaglio space. Consequently, this leads to increased marginal and internal gaps, with the occlusal gap exhibiting the most pronounced effect. Conversely, decreasing the pixel offset in a negative direction (−1) reduces overall crown dimensions, enlarging the intaglio space and improving internal adaptation. The statistically significant trends observed in this study (*p* < 0.05) further confirm the role of pixel offset as a critical factor influencing crown fit. Pixel offset adjustments as a resolution calibration method have been extensively investigated in engineering disciplines [23,24,25,26]. Previous studies in digital imaging systems have explored pixel offset techniques to enhance dimensional accuracy in printed outputs [23,24,25,26]. Similarly, pixel offset has been applied in 3D printing to fine-tune fabrication precision. However, the present study appears to be the first to systematically investigate the optimization of dental prosthesis fit using pixel offset adjustments in DLP 3D printing. While previous studies have primarily focused on evaluating the general fit of resin- and metal-based 3D-printed restorations [43,44], this study introduces pixel offset as a quantitative parameter for optimizing zirconia crown adaptation. These findings offer valuable insights into digital prosthodontics, demonstrating that precise pixel offset adjustments can enhance the clinical accuracy and performance of 3D-printed dental restorations.

### 4.2. Surface Trueness

The findings of this study indicate that pixel offset adjustments in DLP 3D printing significantly influence the surface trueness of zirconia crowns. Increasing the pixel offset resulted in greater overall deviations, with the pixel offset of 1 exhibiting the highest RMS values (57.8 ± 5.2 µm overall, 61.8 ± 4.7 µm external, and 49.5 ± 6.6 µm intaglio). Comparatively, pixel offsets of 0 and −1 showed lower deviation values, with the pixel offset of 0 demonstrating the highest level of surface accuracy (32.8 ± 2.9 µm overall, 35.9 ± 3.7 µm external, and 26.0 ± 3.1 µm intaglio). The observed increase in surface deviation with higher pixel offsets can be attributed to excessive polymerization at the periphery of the printed structure, leading to pronounced overgrowth and positive errors. Conversely, pixel offset −1 exhibited a greater prevalence of negative deviations, likely due to insufficient polymerization in boundary regions.

The impact of pixel offset on surface trueness has not been extensively investigated in previous studies on zirconia 3D printing. However, prior research comparing additive and subtractive manufacturing methods has consistently reported that milled zirconia crowns demonstrate superior surface trueness compared to 3D-printed counterparts [45]. This discrepancy is largely due to the layer-by-layer fabrication process in 3D printing, which inherently introduces stair-stepping effects and variations in material shrinkage [45]. In studies evaluating SLA-printed zirconia crowns, surface deviations ranging from 10 to 50 µm have been reported [29,46], which aligns with the trueness observed in the pixel offset 0 group of the present study. Additionally, investigations into the effects of post-processing techniques, such as sintering and polishing, have demonstrated that deviations introduced during printing can be mitigated to some extent through controlled finishing protocols. However, few studies have explored the role of pixel offset as a modifiable parameter to optimize surface accuracy, highlighting the novelty of the present findings.

Surface trueness is a critical factor in the clinical performance of fixed dental prostheses, as deviations beyond acceptable thresholds may compromise fit accuracy, necessitate extensive post-processing adjustments, or impact the longevity of the restoration [47]. The acceptable deviation range for surface trueness in dental prosthetics has been reported to be between 50 and 100 µm [48], with deviations below 50 µm generally considered optimal. In the present study, the pixel offset of 1 produced surface deviations that exceeded this threshold, indicating a potential risk for compromised clinical performance. In contrast, the pixel offsets of 0 and −1 maintained surface trueness within an acceptable range, suggesting that precise calibration of pixel offset is essential to achieving clinically viable restorations. Furthermore, excessive positive errors observed in the pixel offset 1 group may lead to premature occlusal contacts and increased wear, while excessive negative errors in the pixel offset −1 group may result in insufficient material volume, potentially compromising mechanical strength.

The influence of pixel offset on surface trueness can be explained by its role in controlling dimensional accuracy in the XY plane. As pixel offset increases in a positive direction, the external and internal contours of the printed object expand, leading to an increase in overall crown dimensions and excessive material accumulation at the outermost boundaries. This overgrowth contributes to positive deviations and irregular surface morphology. Conversely, decreasing the pixel offset in a negative direction reduces external dimensions, increasing the likelihood of underbuilt regions and negative deviations. These findings are consistent with research in digital imaging and precision manufacturing [24], where pixel offset adjustments have been used to enhance resolution and reduce geometric distortions. In the field of 3D printing, pixel offset calibration has been explored in polymer-based and metal-based additive manufacturing processes, but its direct application in optimizing ceramic-based 3D printing has been largely unexplored. The present study provides new insights into the role of pixel offset in ceramic 3D printing, demonstrating that controlled adjustments can improve fabrication accuracy and minimize errors in the final restoration.

The results of this study suggest that pixel offset must be carefully optimized to balance surface trueness with overall fit accuracy. While reducing pixel offset improves marginal adaptation, excessive negative pixel offsets may lead to increased negative deviations, potentially compromising the overall integrity of the restoration. Similarly, increasing pixel offset beyond an optimal threshold results in excessive surface irregularities that could affect clinical performance. Therefore, an intermediate pixel offset setting, such as 0, may provide the best balance between fit accuracy and surface trueness. Further investigations are needed to explore the combined effects of pixel offset, layer thickness, and post-processing conditions to establish standardized fabrication protocols for zirconia crowns produced via DLP 3D printing. Future research should also assess the long-term clinical implications of these surface deviations, particularly regarding wear resistance, marginal stability, and patient-reported outcomes.

### 4.3. Correlation Between Intaglio Surface Trueness and Marginal and Internal Fit

The correlation analysis between intaglio surface trueness and the marginal and internal fit of zirconia crowns fabricated using DLP 3D printing revealed statistically significant relationships between specific parameters. A moderate positive correlation was observed between overall fit and mean positive error (PCC = 0.475, *p* = 0.008), indicating that an increase in positive deviations in the intaglio surface was associated with a deterioration in overall fit accuracy. Conversely, overall fit exhibited a weak negative correlation with mean negative error (PCC = −0.390, *p* = 0.033), suggesting that negative deviations contributed to improved adaptation. Similarly, the marginal gap showed a moderate positive correlation with mean positive error (PCC = 0.464, *p* = 0.01) and a weak negative correlation with mean negative error (PCC = −0.396, *p* = 0.03). Among the evaluated parameters, the occlusal gap demonstrated the strongest correlations, exhibiting a moderate positive relationship with mean positive error (PCC = 0.582, *p* < 0.001) and a strong negative correlation with mean negative error (PCC = −0.614, *p* < 0.001). No statistically significant correlations were identified between axial gap and any of the surface trueness parameters (*p* > 0.05). A previous study reported a strong positive correlation between trueness and fitness, indicating that a decrease in trueness (i.e., lower accuracy) is associated with a deterioration in both marginal and internal fit [49].

These findings indicate that surface trueness, particularly in the intaglio region, is a key determinant of the marginal and internal adaptation of zirconia crowns. Higher positive intaglio errors, which represent overbuilt regions, resulted in increased marginal and internal gaps, leading to compromised fit accuracy. Conversely, moderate negative errors facilitated closer adaptation to the prepared tooth structure, improving overall fit. However, excessive negative deviations may also result in insufficient material volume, potentially compromising the mechanical integrity of the restoration.

The correlation between intaglio surface trueness and prosthetic adaptation has significant clinical implications for the fabrication of fixed dental prostheses. Previous studies have highlighted the importance of maintaining minimal surface deviations to ensure optimal fit, with deviations exceeding 50–100 µm associated with increased marginal discrepancies and reduced long-term stability. The findings of this study support these recommendations, as the highest positive errors, observed at a pixel offset of 1, correlated with the largest marginal gaps. In contrast, the negative errors associated with a pixel offset of −1 contributed to improved fit accuracy. While moderate negative deviations may be beneficial for enhancing adaptation, excessive reductions in material volume could lead to structural weaknesses or misalignment during cementation. These findings suggest that careful control of fabrication parameters is necessary to achieve the optimal balance between surface trueness and fit accuracy.

The observed correlations emphasize the critical role of precise parameter optimization in digital dental workflows. Pixel offset adjustments in DLP 3D printing serve as a key determinant of fit accuracy by directly influencing intaglio surface trueness. While increasing the pixel offset results in excessive material buildup and greater positive errors, reducing the pixel offset leads to improved adaptation but must be carefully calibrated to avoid excessive material reduction. In the field of 3D printing, pixel offset calibration has been extensively studied for polymer- and metal-based additive manufacturing processes, but its impact on ceramic-based restorations remains largely unexplored. The present study provides new insights into the role of pixel offset in ceramic 3D printing, demonstrating that controlled adjustments can improve fabrication accuracy and minimize errors in the final restoration.

Further research is warranted to explore the combined effects of pixel offset, printing resolution, and post-processing conditions on the adaptation of 3D-printed zirconia crowns. Additionally, future studies should investigate the influence of occlusal forces, cement space adjustments, and intraoral aging on the long-term clinical performance of restorations with varying surface trueness. The results of this study underscore the importance of precise calibration in digital prosthodontics, highlighting that optimizing pixel offset can enhance both fit accuracy and surface trueness, ultimately leading to improved clinical performance of 3D-printed zirconia restorations.

### 4.4. Limitations of This Study and Future Research Directions

While this study provides valuable insights, several limitations should be noted. First, it was conducted under controlled in vitro conditions that do not fully replicate the complexities of the intraoral environment. Factors such as occlusal forces, variations in cement thickness, and thermal or mechanical aging were not considered. Future research should incorporate these clinical variables to assess long-term performance. Second, the triple-scan protocol used for fit evaluation, while widely applied, has inherent limitations in scan alignment precision. Alternative methods, such as micro-CT analysis, could provide more detailed assessments. Micro-CT scanning offers higher resolution and more accurate three-dimensional reconstructions, which could enhance the evaluation of surface trueness by minimizing potential errors introduced during the scanning and alignment process. Future studies should consider incorporating micro-CT analysis to improve measurement precision and validate the findings of this study. Additionally, this study focused primarily on pixel offset as the key parameter affecting fabrication accuracy. Other factors such as layer thickness, printing resolution, build orientation, and post-processing conditions also influence dimensional accuracy and should be explored in future studies. Moreover, the findings were based on a single DLP 3D printing system and zirconia material, limiting generalizability. Further research should validate these results across different systems and materials. Another important clinical factor that was not considered in this study is the effect of cementation. The marginal and internal fit of a crown may change after cement application due to variations in cement thickness and polymerization shrinkage. Previous studies have shown that cement gaps can influence the long-term retention and stability of restorations. Future research should investigate the interaction between pixel offset settings and cementation processes to better predict clinical outcomes. Furthermore, this study was conducted under static conditions, without accounting for the influence of occlusal forces. In clinical settings, repeated occlusal loading may cause micro-movements in the crown, potentially affecting marginal adaptation and long-term stability. Future studies should incorporate mechanical loading simulations or cyclic fatigue testing to evaluate the impact of occlusal forces on the clinical performance of DLP-printed zirconia crowns. Lastly, while correlation analysis identified significant relationships between surface trueness and fit accuracy, additional studies are needed to examine clinical implications such as crown retention, occlusal adjustments, and patient-reported outcomes. Prospective clinical trials will provide further validation for the practical application of pixel offset adjustments in digital prosthodontics.

## 5. Conclusions

This study investigated the effect of pixel offset adjustments in DLP 3D printing on the marginal and internal fit and surface trueness of zirconia crowns. The results indicate that pixel offset significantly influences both fit accuracy and surface deviations. Higher pixel offsets led to increased marginal and internal discrepancies, with the marginal gap exceeding the clinically recommended threshold of 120 µm at a pixel offset of 1 (114.5 ± 14.6 µm), whereas a pixel offset of −1 (85.5 ± 18.6 µm) remained within the acceptable range. Since a smaller marginal gap is clinically preferable, optimizing pixel offset is essential to improving crown adaptation.

Intaglio surface trueness was also affected, with higher pixel offsets resulting in increased positive errors and lower pixel offsets leading to greater negative errors. Correlation analysis revealed that positive intaglio errors were associated with poor fit, while negative intaglio errors contributed to improved adaptation. The pixel offset of −1 produced greater negative intaglio errors, ultimately leading to enhanced fit accuracy.

These findings highlight the importance of precise pixel offset adjustments in DLP 3D printing to optimize the fabrication of zirconia crowns. Improper pixel offset settings may lead to excessive marginal discrepancies and surface errors, potentially compromising the clinical adaptation and longevity of restorations. Thus, optimizing pixel offset is crucial for achieving clinically acceptable fit and improving the reliability of digitally fabricated prostheses. Further in vivo validation is necessary to confirm the clinical effectiveness and long-term stability of optimized pixel offset settings in DLP 3D printing.

## Figures and Tables

**Table 1:** Chemical composition and physical properties of zirconia material used in this study.

Category	Property	Value
Chemical Composition	Zirconium Dioxide (ZrO_2_)	~94–97%
	Yttrium Oxide (Y_2_O_3_)	~3–5%
	Aluminum Oxide (Al_2_O_3_)	<0.5%
	Hafnium Oxide (HfO_2_)	<0.5%
	Silicon Oxide (SiO_2_)	Trace Amounts
Zirconia Slurry	Solids loading (vol%)	45~54
	Viscosity (Pa·s)	1.8~15
Sintered Zirconia	Theoretical density (g/cm³)	6.05
	Relative density (%)	99.5
	Porosity (%)	0.5
	3-point bending strength (MPa)	600~950

**Table 2:** Comparison of marginal and internal fit of zirconia crowns fabricated using DLP 3D printing with different pixel offset adjustments.

Marginal and Internal Fit	Pixel Offset	Mean (µm)	SD	95% Confidence Interval		Minimum	Maximum	F	*p*
				Lower	Upper				
Overall	1	109.9 ^A^	9.2	103.3	116.5	92.1	120.0	6.732	0.004 *
	0	96.0 ^AB^	15.1	85.3	106.8	74.4	119.1		
	−1	86.5 ^B^	17.4	74.1	99.0	58.0	117.8		
Marginal Gap	1	114.5 ^A^	14.6	104.1	125.1	91.4	145.4	7.427	0.003 *
	0	95.9 ^A^	17.7	83.3	108.6	70.4	129.7		
	−1	85.5 ^B^	18.6	72.3	98.9	54.8	119.8		
Axial Gap	1	96.3	11.4	88.1	104.4	78.4	110.3	1.424	0.258
	0	87.7	12.7	78.6	96.8	68.3	107.8		
	−1	89.3	12.1	80.6	97.9	70.9	111.6		
Occlusal Gap	1	141.9 ^A^	20.2	127.5	156.5	117.5	183.2	16.525	<0.001 *
	0	112.4 ^B^	22.5	96.4	128.6	80.7	148.8		
	−1	80.3 ^C^	28.4	60.1	100.7	31.8	129.1		

* Indicates statistical significance as determined by one-way ANOVA (*p* < 0.05). Uppercase letters denote statistically significant differences between groups based on Tukey’s HSD test, with identical letters indicating no statistically significant difference between groups.

**Table 3:** Comparison of root mean square value of surface trueness of zirconia crowns fabricated using DLP 3D printing with different pixel offset adjustments.

Evaluated Region	Pixel Offset	Mean (µm)	SD	95% Confidence Interval		Minimum	Maximum	F	*p*
				Lower	Upper				
Overall	1	57.8 ^A^	5.2	54.1	61.5	51.6	64.4	102.98	<0.001 *
	0	32.8 ^B^	2.9	30.8	34.9	27.4	36.5		
	−1	36.3 ^B^	4.3	33.3	39.4	31.1	43.0		
External	1	61.8 ^A^	4.7	58.4	65.2	55.9	68.0	125.17	<0.001 *
	0	35.9 ^B^	3.7	33.3	38.5	29.6	41.5		
	−1	34.3 ^B^	4.6	31.0	37.7	28.4	41.7		
Intaglio	1	49.5 ^A^	6.6	44.7	54.3	39.9	59.0	56.493	<0.001 *
	0	26.0 ^B^	3.1	23.9	28.2	22.9	30.6		
	−1	39.6 ^C^	4.5	36.4	42.8	35.1	49.1		

* Indicates statistical significance as determined by one-way ANOVA (*p* < 0.05). Uppercase letters denote statistically significant differences between groups based on Tukey’s HSD test, with identical letters indicating no statistically significant difference between groups.

**Table 4:** Comparison of positive error value of surface trueness of zirconia crowns fabricated using DLP 3D printing with different pixel offset adjustments.

Evaluated Region	Pixel Offset	Mean (µm)	SD	95% Confidence Interval		Minimum	Maximum	F	*p*
				Lower	Upper				
Overall	1	53.5 ^A^	5.1	49.9	57.2	47.4	60.5	179.48	<0.001 *
	0	29.5 ^B^	2.7	27.7	31.5	24.9	33.0		
	−1	23.6 ^C^	3.0	21.6	25.8	18.3	27.0		
External	1	56.9 ^A^	4.6	53.7	60.3	51.0	62.8	198.77	<0.001 *
	0	32.5 ^B^	3.1	30.3	34.8	27.1	37.4		
	−1	24.9 ^C^	3.4	22.5	27.4	19.0	28.8		
Intaglio	1	46.6 ^A^	6.4	42.0	51.2	36.6	55.7	122.10	<0.001 *
	0	22.4 ^B^	3.3	20.1	24.7	18.6	29.5		
	−1	19.1 ^B^	1.9	17.7	20.5	16.1	23.2		

* Indicates statistical significance as determined by one-way ANOVA (*p* < 0.05). Uppercase letters denote statistically significant differences between groups based on Tukey’s HSD test, with identical letters indicating no statistically significant difference between groups.

**Table 5:** Comparison of negative error value of surface trueness of zirconia crowns fabricated using DLP 3D printing with different pixel offset adjustments.

Evaluated Region	Pixel Offset	Mean (µm)	SD	95% Confidence Interval		Minimum	Maximum	F	*p*
				Lower	Upper				
Overall	1	15.6 ^A^	3.4	18.1	13.2	20.2	10.9	69.805	<0.001 *
	0	18.2 ^A^	2.6	20.1	16.4	21.9	13.9		
	−1	32.1 ^B^	3.9	35.0	29.4	39.5	27.4		
External	1	12.9 ^A^	4.0	15.8	10.1	21.3	8.2	48.377	<0.001 *
	0	17.2 ^B^	2.7	19.2	15.3	21.8	13.0		
	−1	28.7 ^C^	4.3	31.8	25.7	38.3	24.1		
Intaglio	1	17.1 ^A^	4.8	20.6	13.6	26.0	10.6	67.976	<0.001 *
	0	19.2 ^A^	3.4	21.7	16.8	26.3	14.5		
	−1	36.6 ^B^	4.0	39.4	33.8	44.7	32.0		

* Indicates statistical significance as determined by one-way ANOVA (*p* < 0.05). Uppercase letters denote statistically significant differences between groups based on Tukey’s HSD test, with identical letters indicating no statistically significant difference between groups.

**Table 6:** Correlation between intaglio surface trueness parameters and marginal and internal fit parameters.

Marginal and Internal Fit Parameters	Pearson Correlation Coefficient (PCC)	Intaglio Surface Trueness Parameters		
		Root Mean Square	Mean Positive Error	Mean Negative Error
Overall fit	PCC	0.218	0.475 **	−0.390 **
	*p*	0.247	0.008 *	0.033 *
Marginal Gap	PCC	0.209	0.464 **	−0.396 **
	*p*	0.268	0.01 *	0.03 *
Axial Gap	PCC	0.207	0.226	−0.001
	*p*	0.272	0.230	0.996
Occlusal Gap	PCC	0.194	0.582 **	−0.614 **
	*p*	0.304	<0.001 *	<0.001 *

* Indicates a statistically significant correlation as determined by the Pearson correlation coefficient (*p* < 0.05). ** A significant correlation denotes a positive or negative relationship between the two variables, with the correlation strength classified as follows: perfect (PCC = −1 or 1), strong (PCC = −0.7 to −0.9 or 0.7 to 0.9), moderate (PCC = −0.4 to −0.6 or 0.4 to 0.6), and weak (PCC = −0.1 to −0.3 or 0.1 to 0.3).

## Data Availability

The data supporting the findings of this study are available from the corresponding author upon reasonable request. Due to privacy and proprietary restrictions, the datasets are not publicly accessible.
